# Digital biomarkers in frontotemporal dementia and related disorders

**DOI:** 10.1002/alz70856_098811

**Published:** 2025-12-24

**Authors:** Rhian S Convery, Lucy L. Russell, Kerala L Adams‐Carr, Jonathan D. Rohrer

**Affiliations:** ^1^ Dementia Research Centre, UCL Queen Square Institute of Neurology, University College London, London, United Kingdom

## Abstract

The Early Detection of Frontotemporal Dementia (EDoF) initiative is focused on identifying sensitive digital biomarkers for the early diagnosis of frontotemporal dementia (FTD) and improving clinical trial readiness. EDoF uses multimodal approaches to assess speech, motor function, behaviour, and cognition. Central to this initiative is the Ignite app, a digital cognitive tool designed to a variety of cognitive domains such as executive function, processing speed, social cognition, semantic knowledge, calculation, visuospatial function, and episodic memory. Validated in healthy controls, the app is now being used in the GENetic Frontotemporal dementia Initiative (GENFI) study in presymptomatic carriers of pathogenic FTD mutations (*C9orf72, GRN, MAPT*) and mutation‐negative family members. Preliminary data shows the Ignite app detects cognitive impairments in asymptomatic mutation carriers with a CDR+NACC‐FTLD‐NM global score of 0, particularly in executive function, processing speed, and social cognition, highlighting its potential as a presymptomatic biomarker. Other tools employed in the EDoF study include digital speech analysis to identify subtle language impairments, Fitbit wristwatches for monitoring activity levels, body‐worn IMU accelerometers for gait analysis, and tapping tests for assessing upper limb motor function. Surface EMG is used to detect muscle fasciculations, aiding early detection of motor symptoms in *C9orf72* mutation carriers. Passive cognitive assessments, such as eye tracking and reaction‐time analysis via the Longevity app, further expand detection capabilities. The EDoF initiative integrates data across multiple modalities to comprehensively measure FTD‐related features and enhance the sensitivity of early detection methods. These efforts aim to develop robust outcome measures for clinical trials and provide a clearer understanding of FTD progression. Early findings underscore the validity of the Ignite app as a promising digital cognitive biomarker. Future work will focus on validating these tools in larger cohorts and establishing frameworks for implementing digital health technologies into routine clinical practice, ultimately supporting earlier diagnosis and improving trial readiness.

